# Prognostic factors in patients with recurrent hepatocellular carcinoma treated with salvage liver transplantation: a single-center study

**DOI:** 10.18632/oncotarget.9040

**Published:** 2016-04-27

**Authors:** Pusen Wang, Hao Li, Baojie Shi, Weitao Que, Chunguang Wang, Junwei Fan, Zhihai Peng, Lin Zhong

**Affiliations:** ^1^ Department of General Surgery, Shanghai General Hospital, Shanghai Jiao Tong University School of Medicine, 200080, Shanghai, China

**Keywords:** hepatocellular carcinoma, liver resection, prognosis, recurrence, salvage liver transplantation

## Abstract

Although salvage liver transplantation (LT) has been widely adopted as a treatment for recurrent hepatocellular carcinoma(HCC), candidate selection criteria have not been established. This single-center study aimed to identify risk factors associated with HCC recurrence and survival following salvage LT. The study included 74 patients treated with salvage LT between October 2001 and February 2013. The median follow-up was 37.2 months after LT. There were 29 cases of HCC recurrence and 31 deaths following LT. Microvascular invasion at the time of liver resection, a time interval to post-LR HCC recurrence of ≤ 12months, an alpha-fetoprotein level at LT greater than 200 ng/mL, and having undergone LT outside of the UCSF criteria were independent risk factors for HCC recurrence after salvage LT. Patients with no more than one risk factor had a 5-year recurrence-free survival rate of 71.2% compared to 15.9% in patients with two or more risk factors. These findings suggest that to avoid post-LT HCC recurrence and a dismal prognosis, patients with no more than one risk factor for recurrence should be given priority for salvage LT. These criteria may improve the outcomes of patients treated with salvage LT and facilitate the effective use of limited organ supplies.

## INTRODUCTION

Hepatocellular carcinoma (HCC) is one of the most common neoplasms and the third leading cause of cancer-related death worldwide [1]. There were an estimated 782,500 new liver cancer cases and 745,500 liver cancer-related deaths in 2012, and HCC accounted for 70–90% of these cases. China accounted for approximately 50% of the total number of cases and deaths [2]. For patients with early-stage HCC, liver transplantation (LT) achieves the best outcomes (5-year survival rate > 80%) because it results in the complete removal of the tumor as well as treats the underlying chronic liver disease [3–5]. However, the shortage of donor organs is a major obstacle, particularly in China, due to the large population base, the donor risks associated with living-donor LT, the high cost of LT, and the associated legal limitations. This ultimately reduces the efficacy of this curative treatment [6].

Salvage LT is a strategy that offers liver resection (LR) followed by LT in patients with tumor recurrence or deteriorating liver function [7]. Previous studies have demonstrated improved survival outcomes in LR, possibly as a result of advanced surgical techniques, patient selection criteria, and postoperative management [8]. Given the shortage of donor organs and the typical progression of HCC (which can cause patients to drop out of the transplant waiting list), LR followed by salvage LT has become widely accepted. Furthermore, salvage LT, defined as LR performed to bridge patients to transplantation and avoid dropout, has been shown to be highly effective, with short- and long-term postoperative survival outcomes comparable to upfront LT [9–15].

HCC recurrence, which is fatal in almost all cases, is still the most important negative predictor of post-LT survival [15]. Over the past few years, substantial efforts have been made to select patients who have a low risk of recurrence, with the aim of curing as many HCC patients as possible [16]. As a result, the Milan, University of California San Francisco (UCSF), and Up-to-Seven (Up-to-7) selection criteria have been proposed. Patients with tumors that meet these criteria have been shown to have a lower rate of post-LT HCC recurrence [3, 17, 18]. Nonetheless, the salvage policy differs from that of primary LT since the former can provide pathological data at LR. This could help predict recurrence, both post-LR [19]and post-salvage LT [20]. However, recurrent cases of HCC exhibited remarkably different clinical behavior despite meeting these criteria [21]. Therefore, the use of only the Milan, UCSF, or Up-to-7 criteria, all of which consider tumor size and number as selection criteria, is not sufficient for salvage LT.

In this retrospective study, we aimed to identify risk factors for HCC recurrence, recurrence-free survival (RFS), and overall survival (OS) after salvage LT in order to improve salvage LT strategies and outcomes, and to help establish proper candidate selection criteria.

## RESULTS

### Patient characteristics

The characteristics of the 74 patients at LR and LT are summarized in Tables 1 and 2, respectively. The participants were predominantly male (n = 68; 91.9%). The underlying liver diseases in the study population were hepatitis B virus (HBV) (n = 68), hepatitis C virus (HCV) (n = 2), and autoimmune hepatitis (n = 4). At the time of the initial LR, 19 patients had an alpha-fetoprotein (AFP) level > 200 ng/mL, 57 patients were within the Milan criteria, 48 patients had an Edmondson grade of 1–2, and microvascular invasion (MVI) was detected in 23 patients. Recurrence occurred > 12 months post-LR in 46 patients and ≤ 12 months post-LR in 28 patients.

**Table 1:** Clinicopathological characteristics of the patients at the initial LR

parameters	
Sex (n)	
Male	68
Female	6
Blood type (n)	
A	29
B	15
AB	7
O	23
Preoperative AFP level (n)	
> 200 ng/mL	19
≤ 200 ng/mL	55
Underlying liver disease (n)	
Hepatitis B	68
Hepatitis C	2
Autoimmune hepatitis	4
HCC status	
Within Milan criteria	57
Outside Milan criteria	17
Edmondson grade (n)	
1-2	48
3-4	26
MVI (n)	
Yes	23
No	51
Time interval to post-LR HCC recurrence (n)	
> 12 months	46
≤ 12 months	28

LR: liver resction; AFP: alphafetoprotein; HCC: hepatocellular carcinoma; MVI: microinvascular invasion.

**Table 2:** Clinicopathological characteristics of the patients at salvage LT

Parameters	
Age	48.9 ± 9.2
Preoperative MELD score	10.8 ± 5.9
Child-Pugh grading (n)	
A	62
B	12
Preoperative AFP level (n)	
> 200 ng/mL	12
≤ 200 ng/mL	62
HCC status (n)	
Within UCSF criteria	57
Outside UCSF criteria	17
TNM staging	
1-2	56
3-4	18
MVI (n)	
Yes	29
No	45
HCC recurrence after Salvage LT (n)	
Yes	29
No	45
Follow-up after LT (months)	37.2 (2.3-81)
Number of deaths (n)	31

LT: liver transplantation; MELD: model for end-stage liver disease; AFP: alphafetoprotein; HCC: hepatocellular carcinoma; UCSF: University of California San Francisco; TNM: tumor node metastasis; MVI: microinvascular invasion.

The mean participant age at the time of salvage LT was 48.9 years, while the mean pre-LT Model for End-Stage Liver Disease (MELD) score was 10.8. Recurrence occurred within the UCSF criteria in 57 cases and outside the criteria in 17 cases. At the time of salvage LT, 62 patients had Child-Pugh grade A, 12 patients had an alpha-fetoprotein (AFP) level > 200 ng/mL, and MVI was detected in 29 patients. The median follow-up after LT was 37.2 (range, 2.3–81) months. A total of 29 cases of HCC recurrence and 31 deaths during LT follow-up were recorded.

### Risk factors for HCC recurrence after salvage LT

Univariate and multivariate analyses were performed to identify risk factors for HCC recurrence after salvage LT (Table 3). On univariate analysis, the strongest pre-LT predictors of recurrence were HCC status outside UCSF criteria at LT with an odds ratio (OR) of 8.33 (95% confidence interval [CI]: 2.36–29.38, *P* < 0.001), and a time interval to post-LR HCC recurrence of ≤ 12months (OR = 5.73;95% CI: 2.05–16.01, *P* = 0.001). The other risk factors significantly associated with HCC recurrence were: advanced Edmondson grade at LR (*P* = 0.016), MVI at LR (*P* = 0.010), AFP level > 200 ng/mL at LT (*P* = 0.009), and advanced tumor node metastasis (TNM) staging at LT (*P* = 0.029).

**Table 3:** Significant risk factors for HCC recurrence after salvage LT (univariate and multivariate analysis)

Variables	Univariate Analysis			Multivariate Analysis		
	OR	95% CI	*p* Value	OR	95% CI	*p* Value
At LR						
Male sex	1.62	0.30-8.61	0.673			
Hepatitis B virus	1.00	0.13-4.44	1.000			
Hepatitis C virus	0.60	0.49-0.72	0.517			
Autoimmune hepatitis	1.59	0.21-11.98	0.642			
Pre-LR AFP level > 200 ng/mL	1.18	0.41-3.40	0.763			
Outside Milan criteria	2.08	0.69-6.25	0.186			
Advanced Edmondson grade	3.31	1.22-8.97	0.016	3.21	0.77-13.51	0.112
MVI presence	3.73	1.33-10.47	0.010	3.71	1.04-13.29	0.044
Post-LR HCC recurrence ≤ 12 months	5.73	2.05-16.01	0.001	4.04	1.23-13.32	0.022
At LT						
Age>50 y	1.11	0.43-2.85	0.83			
Pre-LT MELD score ≥ 15	1.11	0.37-3.36	0.85			
Child-Pugh score ≥ 7	3.85	0.78-20.00	0.098	3.83	0.48-30.30	0.203
Pre-LT AFP level > 200 ng/mL	6.30	1.54-25.83	0.009	6.53	1.17-36.51	0.033
Outside UCSF criteria	8.33	2.36-29.38	< 0.001	6.12	1.39-26.92	0.016
Advanced TNM staging	3.32	1.10-9.98	0.029	1.66	0.36-7.65	0.513

HCC: hepatocellular carcinoma; LT: liver transplantation; LR: liver resction; MVI: microinvascular invasion; MELD: model for end-stage liver disease; AFP: alphafetoprotein; UCSF: University of California San Francisco; TNM: tumor node metastasis; OR: odds ratio; CI: confidence interval.

Variables with *P* values < 0.10 were subjected to multivariate logistic regression analysis, which revealed that MVI at LR (OR = 3.71;95% CI: 1.04–13.29, *P* = 0.044), a time interval to post-LR HCC recurrence of ≤ 12months (OR = 4.04;95% CI: 1.23–13.32, *P* = 0.022), AFP level>200 ng/mL at LT (OR = 6.53;95% CI: 1.17–36.51, *P* = 0.033), and HCC status outside the UCSF criteria at LT (OR = 6.12;95% CI: 1.39–26.92, *P* = 0.016) were independent risk factors for HCC recurrence following salvage LT.

### Prognostic factors affecting RFS and OS after salvage LT

Univariate and multivariate analyses of patients who underwent salvage LT were performed to identify prognostic factors that affected RFS and OS. Univariate analyses using the Kaplan-Meier method and log-rank tests revealed that Edmondson grade 3–4 at LR (*P* = 0.011), the presence of MVI at LR (*P* = 0.003), a time interval to post-LR HCC recurrence of ≤ 12months (*P* < 0.001), TNM stage 3–4 at LT (*P* = 0.018), AFP level > 200 ng/mL at LT (*P* = 0.001), and HCC status outside the UCSF criteria at LT (*P* < 0.001) were identified as risk factors that were significantly associated with RFS after salvage LT. Edmondson grade 3–4 at LR (*P* = 0.004), the presence of MVI at LR (*P* = 0.001), time interval to post-LR HCC recurrence of ≤ 12 months (*P* = 0.001), AFP level>200 ng/mL at LT (*P* = 0.002), HCC status outside the UCSF criteria at LT (*P* = 0.001), and MELD score at LT ≥ 15(*P* = 0.005) were associated with OS. The 1-, 3-, and 5-year survival rates as well as *P* values for the variables are shown in Table 4.

**Table 4:** Variables significantly related to post salvage LT survival (univariate analysis)

Variables	Recurrence-free Survival (%)			HR (95% CI)	*p* Value	Overall Survival (%)			HR (95% CI)	*p* Value
	1 y	3 y	5 y			1 y	3 y	5 y		
Edmondson grade at LR										
1-2	82.9	75.3	65.9	2.51[1.20-5.24]	0.011	85.4	78.3	66.0	2.78[1.36-5.68]	0.004
3-4	79.7	37.3	32.0			84.3	58.7	13.3		
MVI at LR										
Yes	60.9	42.5	37.2	2.84[1.37-5.91]	0.003	73.9	51.0	26.8	3.35[1.61-6.99]	0.001
No	91.5	71.4	62.4			89.9	80.7	58.4		
Post-LR HCC recurrence										
> 12 months	88.7	74.2	70.9	3.85[1.81-8.18]	< 0.001	91.1	83.3	66.5	3.05[1.48-6.29]	0.001
≤ 12 months	70.1	41.3	26.7			75.0	52.5	24.4		
TNM staging at LT										
1-2	85.0	71.5	60.7	2.43[1.14-5.17]	0.018	83.6	75.7	57.6	1.84[0.88-3.86]	0.098
3-4	72.2	34.2	34.2			88.9	58.2	23.3		
AFP level at LT										
> 200 ng/mL	66.7	40.0	13.3	3.38[1.52-7.49]	0.001	75.0	31.3	20.8	3.29[1.48-7.32]	0.002
≤ 200 ng/mL	84.8	66.6	61.5			87.0	79.7	53.5		
HCC status at LT										
Within UCSF criteria	87.4	74.4	66.7	4.47[2.23-9.42]	< 0.001	87.6	79.3	61.5	3.25[1.59-6.66]	0.001
Outside UCSF criteria	62.6	20.9	10.4			76.0	44.3	10.1		
MELD score at LT										
≥ 15						70.6	39.2	29.4	2.79[1.32-5.89]	0.005
< 15						89.4	81.3	52.3		

LT: liver transplantation; LR: liver resction; MVI: microinvascular invasion; HCC: hepatocellular carcinoma; TNM: tumor node metastasis; AFP: alphafetoprotein; UCSF: University of California San Francisco; MELD: model for end-stage liver disease; HR: hazard ratio; CI: confidence interval.

**Figure 1:**
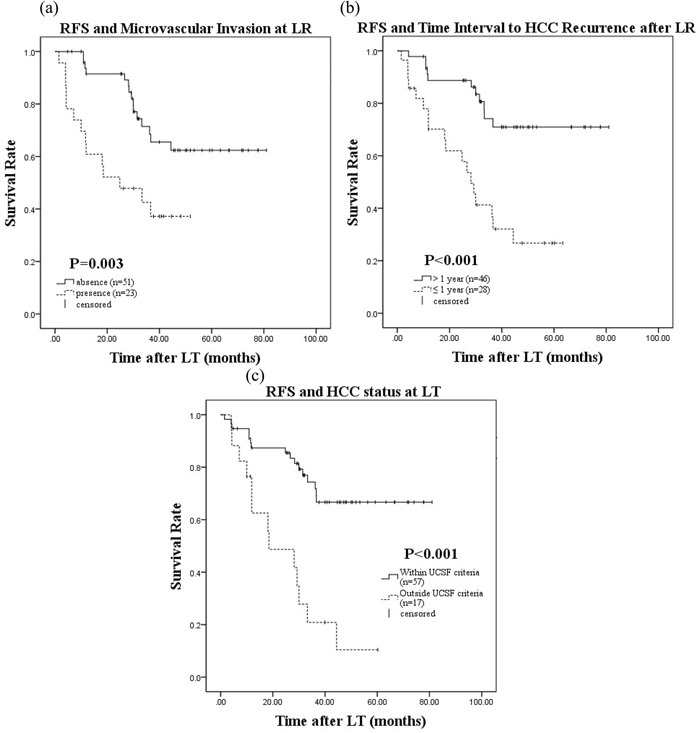
Recurrence-free survival after salvage LT and microvascular invasion at LR a. time interval to HCC recurrence after LR b. HCC status at LT c

**Figure 2:**
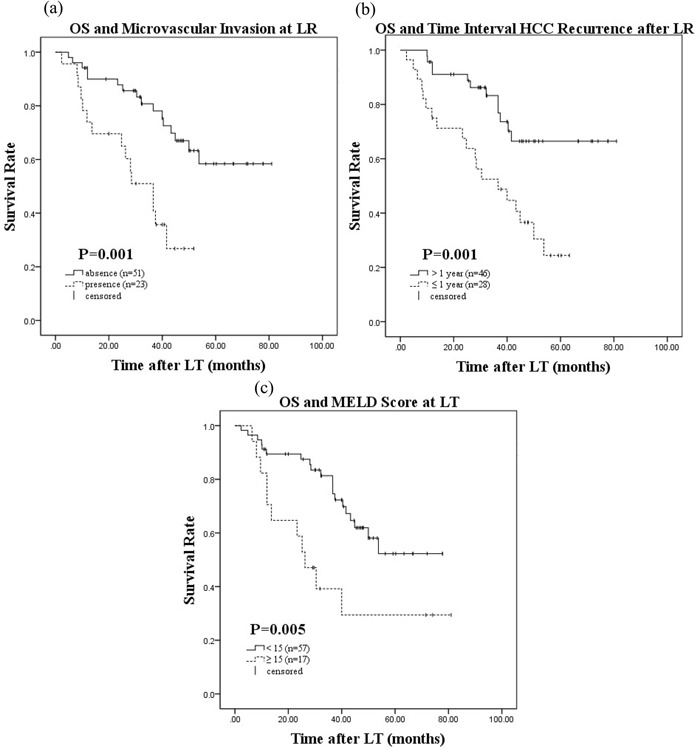
Overall survival after salvage LT and microvascular invasion at LR a. time interval to HCC recurrence after LR b. MELD score at LT c

On multivariate Cox proportional hazard regression analysis, the presence of MVI at LR (hazard ratio [HR] = 3.10;95% CI: 1.44–6.68, *P* = 0.004), time interval to post-LR HCC recurrence of ≤ 12 months(HR = 3.25;95% CI: 1.41–7.49, *P* = 0.006), and HCC status outside the UCSF criteria at LT (HR = 2.68;95% CI: 1.18–6.06, *P* = 0.018) were identified as independent risk factors that affected RFS post-salvage LT. The presence of MVI at LR (HR = 4.47;95% CI: 1.97–10.14, *P* < 0.001), time interval to post-LR HCC recurrence of ≤ 12 months (HR = 3.76;95% CI: 1.78–7.94, *P* = 0.001) and MELD score at LT ≥ 15 affected OS (HR = 4.71, 95% CI: 2.08–10.71, *P* < 0.001) (Table 5). Kaplan-Meier survival curves of RFS and OS according to each risk factor identified on multivariate analysis after salvage LT are shown in Figures 1 and 2, respectively.

**Table 5:** Variables significantly related to post salvage LT survival (multivariate analysis)

	Recurrence-free Survival		Overall Survival	
Variables	HR (95% CI)	*p* Value	HR (95% CI)	*p* Value
MVI presence at LR	3.10[1.44-6.68]	0.004	4.47[1.97-10.14]	< 0.001
Post-LR HCC recurrence ≤ 12 months	3.25[1.41-7.49]	0.006	3.76[1.78-7.94]	0.001
Outside UCSF criteria at LT	2.68[1.18-6.06]	0.018		
MELD score ≥ 15 at LT			4.71[2.08-10.71]	< 0.001

LT: liver transplantation; LR: liver resction; MVI: microinvascular invasion; HCC: hepatocellular carcinoma; UCSF: University of California San Francisco; MELD: model for end-stage liver disease; HR: hazard ratio; CI: confidence interval.

### Prognosis after salvage LT according to number of risk factors

**Figure 3:**
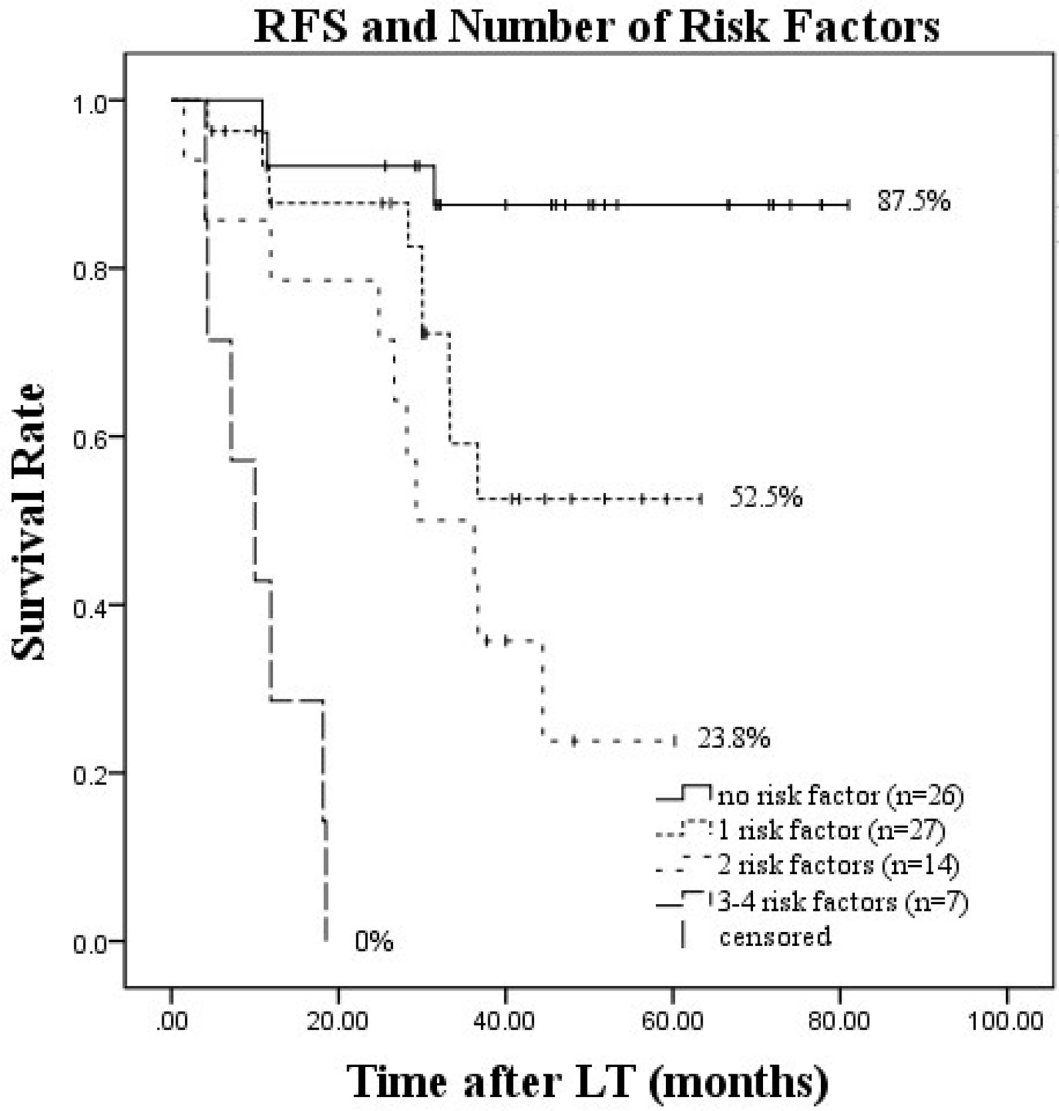
Recurrence-free survival after salvage LT according to number of risk factors

**Figure 4:**
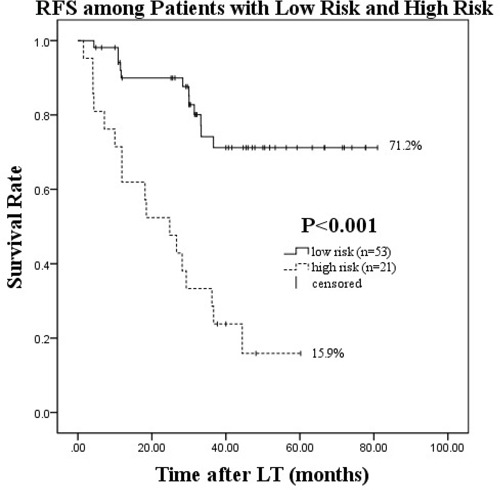
Recurrence-free survival after salvage LT in the low risk (0–1 risk factors) and high risk (2–4 risk factors) groups

On multivariate logistic regression analysis of post-salvage LT HCC recurrence, four independent factors were identified. Patients were divided into four groups according to number of risk factors. The risk of HCC recurrence progressively increased as the number of risk factors increased from 0, 1, or 2 to 3 or 4 as follows: 11.54% (n = 3/26), 33.33% (n = 9/27), 71.43% (n = 10/14), and 100% (n = 7/7), respectively. Kaplan-Meier survival estimates for the four groups showed that when patients with no risk factors were used as a reference, the other three groups had significantly worse RFS (*P* = 0.026, *P* < 0.001, and *P* < 0.001, respectively). The 1-, 3-, and 5-year survival rates and mean RFS are also shown(Table 6, Figure 3). We reported mean survival (Table 6) because there were no more than 50% known deaths during follow-up in patients with 0–1 risk factors, and therefore median survival for these two cohorts was not reached in Kaplan-Meier analysis. Furthermore, two distinct subgroups of patients were defined: those with low risk (0–1 risk factors) and high risk (2–4 risk factors). The Kaplan-Meier survival curves showed a clear distinction between the two groups(*P* < 0.001) (Figure 4).

**Table 6:** Prognosis of patients after salvage LT according to the number of risk factors

Risk Factor Number	Patients (n)	Recurrence n (%)	Recurrence-free Survival (%)			Mean Recurrence-free Survival (95%CI, months)	*p* Value (log-rank test)	
			1 y	3 y	5 y			
0	26	3 (11.54%)	92.1	87.5	87.5	73.24[64.96-81.52]	Ref.	Ref.
1	27	9 (33.33%)	87.7	59.1	52.5	45.74[36.97-54.52]	0.026	
2	14	10 (71.43%)	78.6	42.9	23.8	33.87[23.67-44.06]	< 0.001	< 0.001
3-4	7	7 (100%)	28.6	0.00	0.00	10.59[6.15-15.03]	< 0.001	

LT: liver transplantation; Ref: reference; CI: confidence interval.

## DISCUSSION

We demonstrated that HCC recurrence post-salvage LT was not only dependent on UCSF criteria status, which considers tumor size and number, but was also affected by the biological characteristics of the tumor such as the presence of MVI at initial LR, the time interval to post-LR HCC recurrence, and the pre-LT AFP level. When any of these four independent risk factors were considered, the risk of recurrence increased significantly while RFS decreased compared to patients with no risk factors. Patients at low risk of recurrence had remarkably higher RFS than those at high risk (71.2% vs. 15.9%, respectively). Furthermore, MVI at LR, the time interval to post-LR HCC recurrence, and the MELD score at LT were independent risk factors in addition to the UCSF criteria that affected post-salvage LT survival. Based on these data, we conclude that patients with 0–1 risk factors are better candidates for salvage LT than patients with ≥ 2 risk factors.

Given the donor liver shortage, salvage LT was proposed as an effective way to reduce dropout of patients waiting for a matched donor and delay tumor progression [7]. However, the candidate selection criteria for salvage LT are not well established. As a result of advances in surgical techniques and perioperative management over the past few decades, salvage LT is considered safe for patients deemed to be within the Milan [10], UCSF [14], and Up-to-7 [11] criteria. Additionally, the use of laparoscopic LR has rapidly increased, which has reduced the rate of adhesions that occur near the intestine and omentum, and made subsequent LT much safer when performed by specialist physicians [15]. Furthermore, salvage LT was different when pathologic data were provided at initial LR. Considering all of these factors, more studies are needed to establish the appropriate selection criteria for salvage LT that could supplement the Milan, UCSF, and Up-to-7criteria.

The presence of MVI, an aggressive HCC phenotype, is associated with poor prognosis [22]. In our cohort, 23 patients had MVI at LR compared to 29 at LT. These results were consistent with those of a previous study that reported increased MVI ratios at the time of the second surgery [23]. The presence of MVI at LR was identified as an independent risk factor for recurrence and decreased survival after salvage LT. We observed differences in the MVI results from the two pathological examinations. There were five cases (6.8%) in which MVI status shifted from positive to negative and 11 (14.9%) in which MVI shifted from negative to positive. However, 18 (18/23) patients had MVI at both initial LR and salvage LT, suggestive of a potential correlation in MVI status between at LR and at LT. The presence of MVI in the explanted liver, a strong risk factor for recurrence, was not included in the analysis because it was not clinically relevant to predict post-LT survival at a time point before salvage LT with a variable that only becomes evident post-LT [24].

Lee et al. suggested that patients with microscopic portal vein invasion at initial LR may not be candidates for salvage LT [20]. Additionally, they found that patients with progressive tumors were more likely to have MVI of the portal vein and had a higher incidence of early recurrence ( ≤ 12 months). Another identified risk factor, time interval to post-LR HCC recurrence of ≤ 12months, is reportedly an indication of primary tumor metastasis, while late recurrence is indicative of multi-centric occurrence [25]. Therefore, even in cases of transplantable recurrent HCC(according to the UCSF criteria), there might be a risk of including tumors that originated from metastasis of the primary tumor [20]. Similarly, Hu et al. reported that a time interval to tumor recurrence of < 12 months was an independent predictor for OS in patients who underwent salvage LT [26]. Lee et al. also reported that HCC recurrence within the 8 months following initial LR and a high serum AFP level ( > 200 ng/mL) at salvage LT were independent risk factors for post-salvage LT HCC recurrence [24]. A pre-LT AFP level > 200 ng/mL was also analyzed in our study and determined to be an unfavorable risk factor.

Several previous studies have recommended the use of salvage LT prior to HCC recurrence in patients with unfavorable risk factors [9, 19, 27]. For example, Fuks et al. recommended performing salvage LT before HCC recurrence in patients with ≥ 3 of the five pejorative factors (MVI, satellite nodules, tumor > 3 cm, poorly differentiated tumor, and liver cirrhosis) [9]. However, the main consideration of Fuks et al. was the high risk of nontransplantability after initial LR rather than long-term post-LT patient outcomes. Whether patients with all of these risk factors should undergo salvage LT prior to HCC recurrence is controversial.

Another recent study conducted by Ferrer-Fabrega et al. proposed a waiting time of > 6 months between resection and enlistment for transplant, even in patients at high risk or recurrence (MVI and/or additional nodules or satellites), such that they could identify patients with less aggressive tumors and avoid performing LTs on patients with aggressive tumors (associated with a high risk of post-LT HCC recurrence and decreased survival) [27]. They recommend the use of salvage LT before recurrence in high-risk patients but not in patients with biologically aggressive tumors, in order to avoid disease recurrence with a dismal prognosis post-LT. Nonetheless, our study focused on the time point of recurrence detected after LR to identify the most appropriate candidates for salvage LT considering the limited donor pool. In this regard, our results were consistent with those of Ferrer-Fabrega et al.

This study had several limitations. First, it was retrospective in nature and performed at a single center. Therefore, there were limitations as a result of the analysis of observational data. Our results require further validation by multi-center, prospective studies with larger sample sizes. Second, advances in surgical techniques, improved postoperative management, and more reasonable immunosuppressive therapy would have improved clinical prognosis after the study patient selection period, resulting in heterogeneity among the participants. Finally, the extended period over which patients were selected for inclusion was a limitation of the study.

In conclusion, we aimed to identify appropriate candidates for salvage LT at the time of HCC recurrence detected post-LR. In addition to the UCSF criteria at LT, we demonstrated that the presence of MVI at initial LR, a time interval to post-LR HCC recurrence of ≤ 12months, and a pre-LT AFP level > 200 ng/mL were independent risk factors for post-salvage LT HCC recurrence. Patients with no more than one risk factor had a 5-year RFS rate of 71.2%, while those with > 2 risk factors had a rate of 15.9%. Our findings suggest that the priority of salvage LT should be given to patients with no more than one risk factor to avoid post-LT HCC recurrence and a dismal prognosis. Such a policy might improve salvage LT outcomes and facilitate the effective use of a limited donor organ supply.

## MATERIALS AND METHODS

### Ethical considerations

This study was approved by the Institutional Review Board of the Shanghai General Hospital, Shanghai Jiao Tong University School of Medicine, and was conducted according to the 1964 Helsinki Declaration and its later amendments [28].

### Patients

Between October 2001 and February 2013, a total of 89 HCC patients with a previous history of LR underwent LT for the treatment of HCC recurrence at Shanghai General Hospital, Shanghai Jiao Tong University School of Medicine. All data were obtained from our prospectively maintained LT data base and patient medical records. The HCC diagnosis was confirmed by histopathology of explanted and resected tissue specimens.

Only patients with HCC who underwent salvage LT owing to intrahepatic recurrence after a previous LR were included in the study. The exclusion criteria were: (1) recurrence of other malignancies in addition to HCC such as cholangiocarcinoma (n = 1); (2) tumor thrombosis of the main blood vessel trunk (portal vein and hepatic vein) (n = 2); (3) having undergone LR or LT more than once (n = 3); and (4) a lack of precise pathology data (n = 9).

### Data collection

Patient baseline and clinical data consisting of sex, blood type, underlying liver disease, preoperative AFP level at LR, HCC status according to the Milan criteria at LR, Edmondson grade at LR, presence of MVI at LR, time interval to post-LR HCC recurrence, age at LT, preoperative MELD score at LT [29], Child-Pugh grading at LT [30], preoperative AFP level at LT, HCC status according to the UCSF criteria at LT, TNM staging at LT (according to the International Union Against Cancer/American Joint Committee on Cancer criteria) [31], presence of MVI at LT, HCC recurrence, number of deaths, OS, and RFS were recorded. Surviving patients or those who died of non-HCC causes were censored at the date of their last visit or date of death.

### Tumor surveillance and treatment after LR and LT

After LR and LT, follow-up included ultrasonography, computed tomography, or magnetic resonance imaging scans as well as measurement of serum AFP levels (every 3–4 months for the first 3 years, every 4–6 months for the next 3–5 years, and then every 6–12 months after 5 years) according to the protocol of the Ministry of Health of the People's Republic of China. Recurrence was defined as the appearance of new lesions with HCC features on imaging. Upon detection of recurrence after LR, positron emission tomography/computed tomography, chest computed tomography, or bone scintigraphy was performed to exclude the existence of distant metastases. Post-LT recurrence was treated with locoregional therapies such as radiofrequency ablation, transarterial chemoembolization, radiotherapy, or a combination of these strategies whenever possible. Underlying liver diseases including chronic HBV, HCV, and autoimmune hepatitis were all treated appropriately pre- and post-LT. HBV recurrence was monitored by checking for the presence of HBV surface antigen and HBV DNA in serum at every follow-up visit.

The immunosuppressive regimens used post-LT included either basiliximab or steroids in addition to tacrolimus and mycophenolatemofetil (MMF). Tacrolimus treatment was contraindicated in patients with insufficient renal function (creatinine > 120 μmol/L or creatinine clearance < 40 mL/min). MMF was used in patients with relatively low serum tacrolimus levels as well as in those without pancytopenia (hematocrit > 26% and platelet count > 50,000 cells/mm^3^) [32]. When MMF was administered, tacrolimus was maintained at a lower blood concentration. Liver function as well as the blood concentrations of the immunosuppressants were monitored for dose adjustment purposes.

### Statistical analysis

All of the statistical analyses were performed using the SPSS statistical software, Version 19.0 (SPSS Inc., Chicago, IL, USA). Continuous data were expressed as the mean ± standard deviation (SD) or the median (range), while discrete variables were shown as frequencies. Categorical variables were compared using Pearson's *χ^2^* test or Fisher's exact test, whereas continuous variables were calculated using Student's t-test or the Mann-Whitney test. All variables were dichotomized for analysis. The logistic regression model was applied to investigate post-LT predictors of HCC recurrence. Survival time started at the time of the LT procedure. Survival rates were assessed using Kaplan-Meier analysis and differences between subgroups were compared using the log-rank test. The clinicopathological characteristics of the patients were calculated using the Cox proportional hazard regression model for RFS and OS. The final models were determined by placing all variables with *P* values < 0.10 from the univariate analysis into the multivariate Cox regression analysis. Statistical significance was established as *P* < 0.05.
